# New Keratoconus Risk Factors: A Cross-Sectional Case—Control Study

**DOI:** 10.1155/2022/6605771

**Published:** 2022-09-28

**Authors:** Eloi Debourdeau, Gabriel Planells, Chloe Chamard, David Touboul, Max Villain, Pascal Demoly, Fanny Babeau, Pierre Fournie, Vincent Daien

**Affiliations:** ^1^Department of Ophthalmology, Gui de Chauliac Hospital, Montpellier F-34000, France; ^2^Institute for Neurosciences of Montpellier INM, Universite Montpellier, INSERM, F-34091 Montpellier, France; ^3^National Center of Keratoconus, CHU Bordeaux, Bordeaux F-33000, France; ^4^Centre Hospitalier Universitaire, Maladies Respiratoires, Hôpital Arnaud de Villeneuve, Cedex 5, Montpellier F-34000, France; ^5^National Center of Keratoconus, CHU Toulouse, Toulouse F-31300, France; ^6^The Save Sight Institute, Sydney Medical School, The University of Sydney, Sydney, NSW 2000, Australia

## Abstract

**Purpose:**

To evaluate risk factors associated with keratoconus in a monocentric cross-sectional case-control study.

**Methods:**

This observational study occurred from June 2019 to February 2021 in a university hospital (France). The case group consisted of 195 patients with keratoconus in at least one eye who were followed up by a corneal specialist. The control group consisted of 195 patients without any evidence of keratoconus on slit-lamp examination and corneal topography, who were matched 1 : 1 to controls by age and sex. Data were collected by a self-completed paper questionnaire before the consultation, and a multivariate logistic regression was performed.

**Results:**

Multivariate analysis revealed significant associations of keratoconus with family history (odds ratio [OR] = 22.2, *p* < 0.001), rubbing eyes (OR = 10.9, *p* < 0.001), allergy (any kind) (OR = 3.80, *p* < 0.001), smoking exposure (OR = 2.08, *p*=0.017), and dry eyes (OR = 1.77, *p*=0.045f). The worst eye was associated with the more rubbed eye (*p* < 0.001) and the more pressed eye during the night according to sleeping position (*p* < 0.041).

**Conclusion:**

This study confirmed the association between keratoconus and eye rubbing, family history, and allergy. It highlighted the role of pressure on the eyes during sleep. Other less known risk factors such as dry eyes and smoking exposure should be explored in future studies.

## 1. Introduction

Keratoconus is a bilateral and asymmetric corneal disease [1]. It is characterized by a progressive noninflammatory ectasia, leading to thinning and protrusion of the cornea, which assumes a conical shape. It usually results in vision loss because of refractive errors (irregular astigmatism and myopia) and corneal opacification. It is a public health issue because it usually affects young people and leads to social impairment [2–4]. The disease may progress acutely or be stable. People at a young age seem to be at a major risk factor for disease progression [5].

The disease is multifactorial with genetic [6, 7] and environmental risk factors [8–10]. The genetic contribution is supported by familial aggregation [11, 12], in particular, parental consanguinity [13], and monozygotic twins may have higher ectasia concordance than dizygotic twins [14]. Many genes have been implicated but none alone explains the genesis of the disease [15].

The most recognized environmental risk factor is eye rubbing [16–18]. The repeated mechanical pressure on the cornea causes structural modifications of the collagen, leading to ectasia [19]. Molecular modifications of tears that could explain these histologic changes have been reported in experimental eye rubbings for 60 seconds (increase in levels of metalloproteinases and inflammatory mediators) [20]. This hypothesis is supported by case reports of unilateral or very asymmetric keratoconus when the eye rubbing is unilateral [21–23].

Many ocular surface diseases, leading to itching, have been associated with corneal ectasia. Atopy [24, 25] and allergic conjunctivitis [26, 27] are classical diseases, but many other conditions such as dry eyes [28] and blepharitis [29] have been suspected. Computer vision syndrome [30] is a new entity caused by too much screen time resulting in ocular surface irritation. Studies have found that this syndrome could be associated with keratoconus [28].

Sleeping characteristics in keratoconus patients have been explored. Sleeping position (leading to pressure on the eyes) [23, 28], snoring, and sleep apnea [31, 32] could affect the occurrence of keratoconus, but more investigations are needed. Also, we lack knowledge about the role of the characteristics of eye rubbing in the genesis of keratoconus, such as frequency [11], duration [33], intensity [33], time of the day [23], and part of the hand used for rubbing [28].

This study aimed to more precisely characterize risk factors for keratoconus, especially eye rubbing, in a monocentric population in France.

## 2. Materials and Methods

### 2.1. Ethics Statement

A written informed consent was obtained from each patient in accordance with the 1995 Declaration of Helsinki. The study was approved by the medical ethics committee of the University Hospital of Montpellier (Institutional review board no. 18032020).

### 2.2. Design

This study was a cross-sectional monocentric case-control study performed in Gui de Chauliac University Hospital Center, Montpellier, France, from June 2019 to February 2021.

### 2.3. Study Population

All patients underwent bilateral slit lamp examination and corneal topography using Orbscan II (Placido disc and slit-scanning technology, Bausch and Lomb, Rochester, NY) or Pentacam HR (Scheimpflug imagery, Oculus, Wetzlar, Germany). The primary outcome (keratoconus diagnosis) was determined by two corneal specialists unaware of the patient's risk factors.

The inclusion criterion for the keratoconus group was clinical or topographic keratoconus in at least one eye. Patients who already had a corneal graft for keratoconus were also included.

Except for grafted eyes, each eye with keratoconus was classified from stage I to stage IV using the modified Amsler–Krumeich classification [34, 35]. For each case, we defined which eye was the worst according to the classification. For cases with grafted eyes with no previous topography available in the medical file, we defined the first eye operated as the worst.

The inclusion criterion for the control group was no clinical or topographic keratoconus in any eye. The control group was sex and age-matched, with a 2-year tolerance, to the case. Controls were patients consulting an ophthalmologist in our center for reasons other than keratoconus mostly for a routine examination.

Exclusion criteria were doubtful topography or slit lamp examination or refusing participation.

### 2.4. Data Source

Each enrolled patient had to complete a paper questionnaire in the waiting room before an ophthalmologist consultation. This questionnaire was created by the National Center of Keratoconus and is currently subjected to a validation study.

The data concerned demographics (age, sex, social status, and dominant hand), medical history (time since keratoconus diagnosis, family history of keratoconus, allergies, eye diseases, dry eye based on OSDI questionnaire), eye rubbing characteristics (frequency, part of the hand used, duration, intensity, time of the day, causes, noise, etc.), sleeping characteristics (regularity, position, snoring or apnea), and environmental exposure (screen time, weather, tobacco exposure, animals, irritant products, etc.).

### 2.5. Statistical Analysis

Continuous statistics are reported as mean ± SD and median (range) for continuous variables. Categorical variables are reported as numbers (percentages). The nonparametric MannWhitney test was used to compare continuous variables and the chi-square test (or Fisher's exact test as appropriate) to compare categorical variables.

Potential factors associated with keratoconus were compared on univariate analysis (chi-square test or Fisher's exact test). Thereafter, a multivariate logistic model was built based on selected parameters from the univariate analysis (level of significance set at *p* < 0.20 for selection). In addition, three procedures for selecting variables (forward, backward, and stepwise) by the minimization of Akaike's information criterion were used to obtain the most appropriate logistic regression model. The absence of multicollinearity has been verified by using the variance inflation factor. Odds ratios (ORs) and 95% confidence intervals (CIs) were estimated. *p* < 0.05 was considered statistically significant, and all statistical tests were two-sided. Statistical analyses were performed with *R* 3.6.1 (Vienna, Austria). No imputation was used for missing values because less than 1% were missing.

## 3. Results

### 3.1. Participants (Figure 1 and Table 1)

We included 195 patients with keratoconus who were followed up by a corneal specialist, and 195 healthy patients were matched on age and sex. Most participants were men (67%), which is similar to the literature [36]. The mean age was 32 ± 12 years. The mean time since diagnosis was 7 ± 9 years.

In the case population, the mean maximum keratometry (*K*_max_) was 49.1 ± 5 diopters and the thinnest pachymetry was 441 ± 66 microns. In the control population, these parameters were 43.7 ± 1.5 diopters and 543 ± 37 microns. Eyes with corneal grafts were excluded from these analyses. These parameters were all statistically different (*p* < 0.001) between the two groups.

Of the 390 eyes with keratoconus enrolled, 152 (39%) eyes were at stage I, 85 (22%) at stage II, 75 (19%) at stage III, 35 (9%) at stage IV, and 41 (11%) with corneal graft according to the modified Amsler–Krumeich classification.

### 3.2. Risk Factors of Keratoconus (Tables 2 and 3 and Figure 2)

The first univariate analysis concerned the whole population of cases and controls. In total, 164 (84%) cases and 66 (34%) controls had a history of eye rubbing (*p* < 0.001) and 20% (*n* = 39) of cases had a family history of keratoconus (*p* < 0.001). Eye dryness was significantly associated with keratoconus (*p* < 0.001). The only sleeping position associated with keratoconus was the contact of the hand or the forearm with the eyes (*p*=0.010). Allergies, in particular asthma and allergic conjunctivitis, were associated with the disease (*p* < 0.001). Also, cases were more frequently exposed to smoke (tobacco or cannabis) than controls (*p* < 0.015).

The parameters selected from univariate analysis for a multivariate logistic model were family history, eye rubbing history, allergy (any kind), dry eyes, smoking exposure, screen time, hand or forearm contact with the eyes during sleeping and snoring, or obstructive sleep apnea (OSA).

The multivariate model confirmed the association of keratoconus with family history (OR = 22.2, 95% CI: 5.88–148; *p* < 0.001), history of rubbing eyes (OR = 10.9, 95% CI: 6.35–19.3; *p* < 0.001), allergy (any kind) (OR = 3.80, 95% CI: 2.26–6.54; *p* < 0.001), smoking exposure (OR = 2.08, 95% CI: 1.15–3.85; *p*=0.017) ,and dry eyes (OR = 1.77, 95% CI: 1.02–3.09; *p*=0.045).

### 3.3. Analysis of Rubbing Characteristics (Table 4 and Figure 3)

We analyzed rubbing characteristics in the population of people who rubbed their eyes (164 cases and 66 controls). On univariate analysis of the eye rubbing population, keratoconus was associated with frequency of rubbing “often, 1–10 times a day” (OR = 8.12, 95% CI: 4.13–16.6; *p* < 0.001) and “very often ≥10 times a day” (OR = 52.1, 95% CI: 10.4–951; *p* < 0.001), duration “≥15 sec” (OR = 2.74, 95% CI: 1.16–7.57; *p*=0.032), “hard” intensity of rubbing (OR = 3.06, 95% CI: 1.28–8.20; *p*=0.017), and rubbing noise (OR = 1.97, 95% CI: 1.06–3.77; *p*=0.036). Other associated factors were using knuckles for rubbing (OR = 2.46, 95% CI: 1.35–4.59; *p*=0.004) and the back of the hand (OR = 2.97, 95% CI: 1.19–9.05; *p*=0.032) but index fingertips were less often used (compared to other types of friction) as compared with controls (OR = 0.34, 95% CI: 0.18–0.62; *p* < 0.001).

On multivariate analysis, keratoconus remained associated with rubbing “often, 1–10 times a day” (OR = 9.48, 95% CI: 4.62–20.6; *p* < 0.001) and “very often ≥10 times a day” (OR = 57.3, 95% CI: 11.0–1,059; *p* < 0.001). The only way of rubbing significantly associated with multivariate analysis was protective: “use of fingertips” (OR = 0.27, 95% CI: 0.13–0.55; *p* < 0.001).

### 3.4. “More Advanced Side” Analysis (Table 5)

The side of the worst eye was not linked to the dominant hand used for rubbing (*p*=0.43) but was associated with the preferential eye rubbed (*p* < 0.001) and the side with more mechanical pressure during the night according to the sleeping position (*p*=0.041).

## 4. Discussion

The main point of our study is that it supports the mechanical etiology of keratoconus. According to the literature, we found a strong association between eye rubbing and multivariate analysis [11, 28, 36, 37]. The association between the preferential eye rubbed and the worst diseased eye confirms a strong link. New risk factors have been described: tobacco, probably due to the dryness and the induced rubbing and the way of rubbing the eye. Using knuckles and the back of the hand for rubbing was associated with keratoconus, with less use of the index fingertips for rubbing as compared with controls; knuckles and the back of the hand expose the eye to harder mechanical power by direct bone contact. The main rubbing characteristic leading to keratoconus in the eye rubbing population was high frequency rather than intensity or duration of rubbing.

The worst diseased eye was associated with more eye rubbing and more pressed eyes during the night, according to the sleeping position. Mazharian et al. [23] also found that patients with unilateral or highly asymmetric keratoconus exhibited homolateral eye rubbing and slept on the same side.

Concerning the sleeping position in the total population, the only statistically positive result was contact with the hand or forearm on the eyes during sleep on univariate analysis only. We did not confirm any correlation with the prone, supine, and side sleep positions or screen time as found in the Moran et al. study [28], nor any already suspected associations with sleep apnea [31, 32], perhaps because of a lack of controls included. The sleeping position could be a risk factor for the pathogenesis of keratoconus itself or for the progression of the disease. The genesis of keratoconus is linked to an anatomical predisposition to thin corneas that are less resistant to mechanical stress. Keratoconus, therefore, could only occur in certain patients who have a predisposition and who apply a mechanical constraint on their eyes during sleep or eye rubbing.

Also, on multivariable analysis, we confirmed classical known associations such as family history [11, 12, 36], dry eyes [28], and allergy [38] at rates similar to the literature.

A 2010 meta-analysis considered that the most important risk factors for keratoconus were eye rubbing, positive family history, allergy, asthma, and eczema [9]. The latter factor was the only one not found in our study, perhaps because we analyzed “skin allergies” in general and not eczema in particular. In addition, allergic conjunctivitis, which is a classical risk and disease severity factor [26], was also positive in our univariate analysis (*p* < 0.001).

We found an association between smoking and keratoconus but other studies usually did not [36, 39]. An Australian study in 2021 found a correlation between severity and smoking cigarettes on univariate analysis, which was not confirmed in the multivariate model [40]. Different hypotheses may explain the association between smoking and keratoconus. The dysfunction of the meibomian glands and the dry syndrome induced by smoking could increase friction. The increase in rubbing could also be explained by the personality type of anxiety more frequent in this population. Eye rubbing would decrease the stress felt by the patient via the stimulation of the Vagus *X* nerve by the oculocardiac reflex.

We did not explain this association and other studies should be conducted to confirm this.

Our study has some weaknesses and is prone to bias. First, the data were obtained from a questionnaire. Hence, there is a risk of reporting bias depending on the precision of the questions, the knowledge of patients, and their willingness to cooperate. For example, the fact that cases were aware of the association between keratoconus and eye rubbing may have influenced their response. Keratoconus is an acquired disorder, and we selected our control group with normal topographic and clinical criteria at the time of inclusion. Because of no follow-up, some controls could have exhibited keratoconus during the study, which could imply selection bias. We controlled that bias by excluding all patients with doubtful corneal topography.

## 5. Conclusion

Our study confirmed the most important risk factors for keratoconus: eye rubbing, family history, and allergy. It allows for more understanding of the eyes rubbing habits of our patients by detailing many of the rubbing characteristics associated with the genesis of corneal ectasia. We also found that the sleeping position could play a key role in the pathophysiology of keratoconus. We recommend that all patients be systematically screened for inappropriate sleeping positions to identify the use of night-time eye protection if necessary. These elements are crucial in care because we can use them to advise our patients and help them avoid their habits. Indeed, helping people to stop eye rubbing could be an effective treatment on its own to stop the progression of ectasia. In addition, it can be a target for primary prevention by educating the population to not adopt these behaviors.

## Figures and Tables

**Figure 1:**
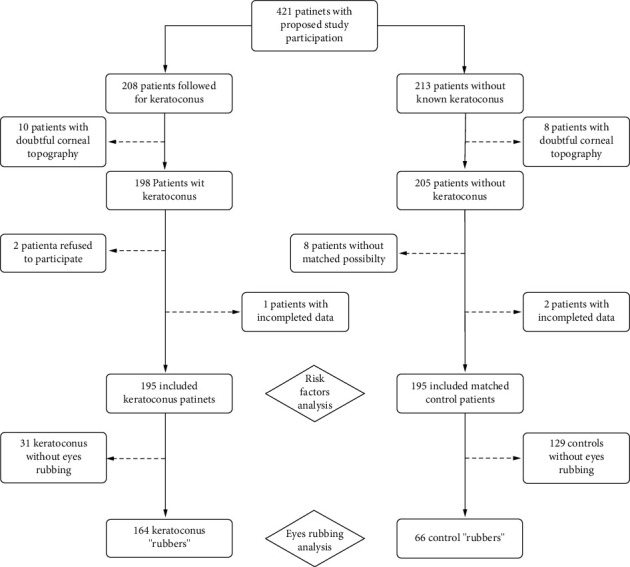
Flow chart.

**Figure 2:**
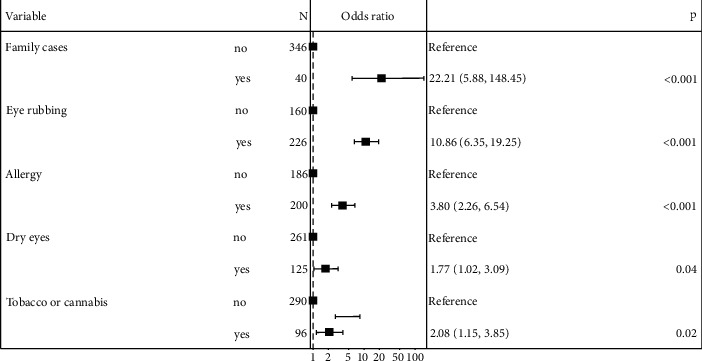
Forest plot of multivariate analysis of risk factors in the general population. Graphical representation of the odds ratio of the risk factors of keratoconus in the general population from the multivariate linear regression.

**Figure 3:**
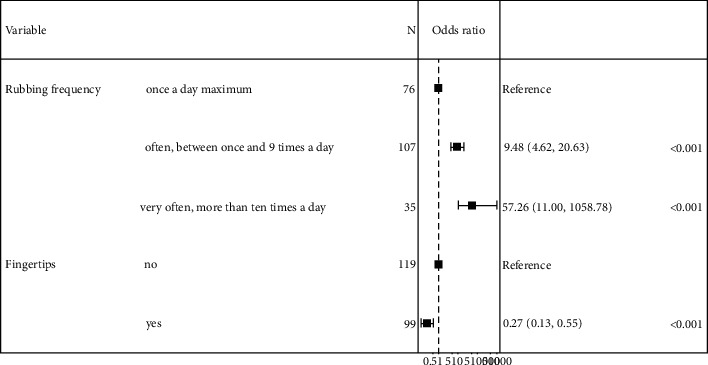
Forest plot of multivariate analysis of risk factors in the eye rubbing population. Graphical representation of the odds ratio of the risk factors of keratoconus in the eye rubbing population from the multivariate linear regression.

**Table 1:** Population characteristics.

	Cases, *N* = 195			Controls, *N* = 195	*p* value^1^
Sex					>0.99
Men	130 (67)			130 (67)	
Women	65 (33)			65 (33)	

Age	32 ± 12; 30 (11–80)			32 ± 12; 30 (10–79)	0.93
Keratometry, diopters					
Kmax (mean right and left)	49.1 ± 5.0; 48.2 (22.0–68.4)			43.7 ± 1.5; 43.6 (37.8–47.6)	**<0.001**
Missing	9			0	
Kmax right	49.3 ± 5.1; 48.2 (41.7–71.3)			43.7 ± 1.6; 43.8 (37.4–47.9)	**<0.001**
Missing	21			0	
Kmax left	48.7 ± 5.1; 47.7 (40.4–74.2)			43.6 ± 1.6; 43.7 (38.2–47.4)	**<0.001**
Missing	22			0	

Pachymetry, microns					
Thinnest (mean right and left)	441 ± 66; 452 (117–572)			543 ± 37; 544 (385–620)	**<0.001**
Missing	10			0	
Thinnest right	444 ± 69; 458 (117–572)			543 ± 37; 544 (380–632)	**<0.001**
Missing	22			0	
Thinnest left	444 ± 70; 458 (226–582)			542 ± 41; 545 (269–622)	**<0.001**
Missing	21			0	

Amsler–krumeich classification	Right	Left	Both		
1	73 (38)	79 (41)	152 (39)		
2	43 (22)	42 (22)	85 (22)		
3	36 (19)	39 (20)	75 (19)		
4	22 (11)	13 (6.7)	35 (9)		
Graft	20 (10)	21 (11)	41 (11)		

Time since diagnosis (years)	7 ± 9; 4 (0–50)				
Intracorneal rings	13 (6.7)				
Cross-linking	53 (27)				

Data are (%) or mean (SD)/median (range); the ^1^chi-square test; and the Wilcoxon rank sum test.

**Table 2:** Risk factors of keratoconus and univariate analysis.

	Cases, *N* = 195	Controls, *N* = 195	*p* value^1^
Rubbing eyes			**<0.001**
Active	108 (55)	66 (34)	
Never	31 (16)	129 (66)	
Past: stopped	56 (29)	0 (0)	
Eye rubbing history (past or active)	164 (84)	66 (34)	**<0.001**

Family history	39 (20)	2 (1.0)	**<0.001**
Dry eyes	81 (42)	19 (9.7)	**<0.001**
History of eyes irritative acnea treatment (retinoid)	6 (3.1)	7 (3.6)	0.78
Screen time per day			0.10
<4 hr	82 (42)	98 (50)	
≥4 hr	113 (58)	97 (50)	

Sleeping data			
Snoring or OSA	41 (21)	27 (14)	0.065
Missing	0	1	
Regular sleep	112 (61)	111 (57)	0.44
Missing	11	0	
On the back	50 (26)	47 (24)	0.73
On the side	131 (67)	122 (63)	0.34
Head in the pillow	44 (23)	51 (26)	0.41
Hand or forearm on the eye	20 (10)	7 (3.6)	**0.010**

Exposure data			
Dry condition or air conditioning	46 (24)	61 (31)	0.083
Missing	0	1	
Smoking (tobacco or cannabis)	59 (30)	38 (20)	**0.015**
Missing	0	1	
Animals	61 (31)	68 (35)	0.43
Missing	0	1	

Allergy data			
Allergy (any kind)	124 (64)	78 (40)	**<0.001**
Missing	2	0	
Asthma	39 (20)	15 (7.7)	**<0.001**
Skin allergies	29 (15)	23 (12)	0.37
Allergic rhinitis or hay fever	53 (27)	40 (21)	0.12
Allergic conjunctivitis	78 (40)	26 (13)	**<0.001**
Angioedema	7 (3.6)	1 (0.5)	0.068
Drug allergies	12 (6.2)	19 (9.7)	0.19
Food allergies	15 (7.7)	10 (5.1)	0.30

OSA = obstructive sleep apnea. Data are *n* (%); the ^1^chi-square test; and the Fisher's exact test.

**Table 3:** Risk factors of keratoconus and multivariate logistic regression.

Characteristic	Univariate analysis			Multivariate analysis		
	OR	95% CI	*p* value	OR	95% CI	*p* value
Family history						
No	—	—		—	—	
Yes	23.4	7.02–145	**<0.001**	22.2	5.88–148	**<0.001**

Eye rubbing history						
No	—	—		—	—	
Yes	10.5	6.54–17.4	**<0.001**	10.9	6.35–19.3	**<0.001**

Allergy (any kind)						
No	—	—		—	—	
Yes	2.77	1.84–4.20	**<0.001**	3.80	2.26–6.54	**<0.001**

Dry eyes						
No	—	—		—	—	
Yes	2.45	1.58–3.83	**<0.001**	1.77	1.02–3.09	**0.045**

Smoking exposure (tobacco or cannabis)						
No	—	—		—	—	
Yes	1.86	1.16–2.99	**0.010**	2.08	1.15–3.85	**0.017**

Screen time per day						
<4 hr	—	—		—	—	
≥4 hr	1.43	0.96–2.13	0.083	1.54	0.91–2.61	0.11

Hand or forearm on the eye (sleeping position)						
No	—	—				
Yes	3.04	1.31–7.90	**0.014**			

Snoring or OSA						
No	—	—				
Yes	1.61	0.95–2.77	0.082			

CI = confidence interval, OR = odds ratio, and OSA = obstructive sleep apnea. Data are *n* (%) or odds ratio (OR) and 95% confidence interval (CI). Odds ratios were calculated by univariate and multivariate logistic regression. The multivariate model was created with the variables with *p* < 0.20 in the univariate analysis with a method of eliminating variables by using the corrected Akaike information criterion. A generalized variance inflation factor was used to check for multicollinearity.

**Table 4:** Risk factors of keratoconus in the eye rubbing population and multivariate logistic regression.

Characteristic	Cases *N* = 164	Controls *N* = 66	Univariate analysis			Multivariate analysis		
			OR	95% CI	*p* value	OR	95% CI	*p* value
Rubbing frequency								
Once/day maximum	97 (59)	17 (26)	—	—		—	—	
Often, 1–10 times/day	32 (20)	48 (73)	8.12	4.13–16.6	**<0.001**	9.48	4.62–20.6	**<0.001**
Very often, >10 times/day	35 (21)	1 (1.5)	52.1	10.4–951	**<0.001**	57.3	11.0–1,059	**<0.001**

Rubbing duration								
<15 sec	34 (22)	6 (9.2)	—	—				
≥15 sec	121 (78)	59 (91)	2.74	1.16–7.57	**0.032**			

Intensity of rubbing								
Superficial	59 (36)	32 (48)	—	—				
Moderate	62 (38)	27 (41)	1.15	0.61–2.18	0.67			
Hard	43 (26)	7 (11)	3.06	1.28–8.20	**0.017**			

Rubbing noise								
No	92 (56)	48 (73)	—	—				
Yes	71 (44)	18 (27)	1.97	1.06–3.77	**0.036**			

Type of rubbing								

Index fingertips								
No	103 (63)	24 (36)	—	—		—	—	
Yes	61 (37)	41 (63)	0.34	0.18–0.62	**<0.001**	0.27	0.13–0.55	**<0.001**

The palm of the hand								
No	138 (84)	54 (82)	—	—				
Yes	26 (16)	11 (17)	0.89	0.41–2.01	0.77			

Knuckles								
No	73 (45)	44 (67)	—	—				
Yes	91 (55)	21 (32)	2.46	1.35–4.59	**0.004**			

Thumb and index clamp								
No	141 (86)	56 (85)	—	—				
Yes	23 (14)	9 (14)	0.91	0.40–2.22	0.83			

Back of the hand								
No	132 (80)	59 (89)	—	—				
Yes	32 (20)	6 (9)	2.97	1.19–9.05	**0.032**			

OR = odds ratio and CI = confidence interval. Data are *n* (%) or odds ratio (OR) and 95% confidence interval (CI). Odds ratios were calculated by univariate and multivariate logistic regression. The multivariate model was created with the variables with *p* < 0.20 in the univariate analysis with a method of eliminating variables by using the corrected Akaike information criterion. A generalized variance inflation factor was used to check for multicollinearity.

**Table 5:** Analysis of eye for more advanced disease.

Laterality	Eye with more advanced disease		*p* value^1^
	Left, *N* = 88	Right, *N* = 107	
Dominant hand			0.43
Left	12 (14)	19 (18)	
Right	76 (86)	88 (82)	

Preferential eye rubbed			**<0.001**
No preferential rubbing	59 (67)	65 (61)	
Left	20 (23)	10 (9.3)	
Right	9 (10)	32 (30)	

Press on the eye during sleeping relative to the sleeping position			**0.041**
Both or none	59 (67)	60 (56)	
Left	14 (16)	12 (11)	
Right	15 (17)	35 (33)	

Data are *n* (%). ^1^ the chi-square test.

## Data Availability

The dataset analyzed during the current study is available from the corresponding author on reasonable request.
